# Crossing Borders for Science

**DOI:** 10.1371/journal.pcbi.1003519

**Published:** 2014-03-27

**Authors:** Sebastian J. Schultheiss, Joshua SungWoo Yang, Wataru Iwasaki, Shu-Hsi Lin, Angela Jean, Magali Michaut

**Affiliations:** 1 Computomics Molecular Data Analysis, Tuebingen, Germany; 2 Department of Physiology, Laboratory of System Medicine, School of Medicine, Ajou University, Suwon, Republic of Korea; 3 Atmosphere and Ocean Research Institute, University of Tokyo, Kashiwa, Chiba, Japan; 4 Biodiversity Research Center, Academia Sinica, Taipei, Taiwan; 5 School of Medicine, University of Queensland, Brisbane, Queensland, Australia; 6 Computational Cancer Biology, Netherlands Cancer Institute, Amsterdam, Netherlands; Princeton University, United States of America

## Abstract

Exchanging ideas with like-minded, enthusiastic people interested in the same topic is crucial for the advancement of a scientist's career. Several Regional Student Groups (RSGs) of the International Society for Computational Biology (ISCB) Student Council have cooperated in the last six years to organize scientific workshops and conferences. With motivated students, it is possible to create a memorable event for fellow scientists; in doing so, the organizers gain valuable experiences. While collaborating across borders and time zones can be difficult, feedback from event organizers was always positive. When limited resources are juxtaposed with great ideas and a network of contacts, the outcome is always an amazing experience, despite organizers being separated geographically across different countries.

## Introduction

Science is connected internationally and is improved through collaboration and networking among peers [1]–[4]. Regional Student Groups (RSGs) of the International Society for Computational Biology (ISCB) Student Council promote interaction among students within the same countries or regions in the fields of bioinformatics and computational biology [5]. The number of RSGs has grown dramatically since their establishment a few years ago, and there were more than 25 RSGs active around the world in 2013 [6]. RSG activities often take place at a local level: members carry out various activities with colleagues from the same region. At the same time, many RSGs also maintain contacts beyond country boundaries, developing computational biology communities to further communication.

These international contacts lead to diverse activities. For example, members from different RSGs may hold teleconferences to exchange information on their recent activities; such communication is important and productive for exploring possible further collaborations. However, in this article, we focus on (i) activities where members from different RSGs meet face-to-face and (ii) meetings that contain a high amount of scientific or educational content. Examples of such international collaborations include scientific conferences, programming workshops, and special symposia auxiliary to international conferences.

A major concern for young students and researchers is the ability to initiate and sustain a fruitful exchange about their research with peers. Activities conducted by multiple RSGs facilitate successful research exchanges by promoting interaction with field experts and participants from all over the world. As events organized by the RSGs focus on the next generation of scientists across countries, they provide opportunities for early-career scientists to connect with leading invited speakers and experienced peers.

In addition to the benefit of increased scientific knowledge that RSG activities provide, facilitating networking and information sharing about research life in other countries are also highlights of such events. When young scientists from around the world get to know each other, they can overcome cultural and geographical barriers to initiate exchange programs for short visits to different laboratories, seek doctoral or post-doctoral positions abroad, and even form practical scientific collaborations. Early international exchanges not only boost young researchers' confidence in pursuing science, but also motivate them to sharpen professional abilities and soft skills, including language, communication, and presentation skills. Here, we highlight three examples of such events.

## Examples of International Events

### Asian Young Researchers' Conference on Computational and Omics Biology (AYRCOB)

During AYRCOB, colleagues from five Asian countries come together to promote exchange among students and young researchers within Asia. Since 2008, AYRCOB has been held seven times in various locations: Hsinchu (Republic of China Taiwan), Tokyo (Japan), Tainan (Republic of China Taiwan), Singapore, Daejeon (Republic of Korea), Shenzhen (People's Republic of China) in collaboration with the ISCB-Asia conference, and most recently, in September 2013, in Tokyo (Japan).

AYRCOB has grown dramatically and has become a unique and outstanding conference for young students in Asia. It is a truly international conference, where the entire three-day program is organized by the students. Attendees have the opportunity to present their research in the form of talks and posters to over 100 peers from more than ten Asian countries. The conference provides participants with invaluable networking opportunities across Asia. As the conference grew rapidly, selections for oral presentations have become extremely competitive, with an acceptance rate of 20% in 2012. In addition, distinguished senior researchers give invited lectures on their exciting research topics.

The conference organizers communicate using a mailing list, a wiki site, and Skype calls. While the outcome of the planning process is important, the process itself also fulfills an objective of AYRCOB: to provide the experience of organizing an international conference with colleagues across several countries. Sometimes, enduring friendships develop among the organizers.

Inevitably, many unforeseen challenges can arise when organizing a conference this large. To overcome these, previous organizers have formalized the decision-making process and distributed responsibilities among themselves to make the workload bearable. AYRCOB has introduced specialized groups, such as the program, budget, local, proceedings, and public relations committees, modeled after similar popular international conferences, such as the ISCB Student Council Symposia, which have a comparable organizational structure.

In recognition of the outstanding achievements of AYRCOB, the Dean Prize of the Graduate School of Frontier Sciences at the University of Tokyo was awarded to the organizers in 2009 and 2011. Additionally, the fifth AYRCOB in 2011 was featured in Korean and Taiwan news media.

### Python Programming Workshop

Recognizing the growing demand for hands-on, tool-oriented training in Python for computational biologists, RSG Germany and RSG Poland organized a compact three-day workshop on using Python in bioinformatics. With the appeal of an international event with motivated students, the RSGs were able to invite three experts on different aspects of (Bio)Python as keynote lecturers to a beautiful and affordable location in the Polish mountains. All participants were invited either to present insights into their current research in impromptu three-minute “lightning” talks or to formulate a problem from their work that was then put forth as a challenge to the invited speakers and the audience. The practical, day-to-day tips from the experts on debugging techniques or software project planning made the workshop an interesting experience even for advanced participants. The location was chosen with great care to ensure easy and equidistant travel for all participants, to facilitate hosting the invited speakers for the entire duration of the meeting, and to allow attendees of the meeting to seek the opinions of the experts throughout the three days. The organizers had invaluable support from faculty advisors of the RSGs, creating an event that was memorable for all participants. This is not an event that a single RSG would have been able to accomplish. Obtaining the necessary funds, finding the perfect location and creating enough “draw” for so many high-quality speakers and such a high number of attendees took a cross-border effort.

### European Student Council Symposium (ESCS)

After several RSGs started in Europe (Netherlands, France, Germany, and Poland), the Student Council initiated a European RSG with the purpose to foster interactions and exchanges between RSGs in Europe and to promote the creation of new RSGs. Since then, RSGs have been established in Spain, Portugal, Italy, Belgium, and Turkey. This umbrella group creates opportunities for all European RSGs to collaborate and organize events together. One of the main events was held in association with the European Conference on Computational Biology (ECCB) in 2010: the first European Student Council Symposium (ESCS). This symposium brings more students to the ECCB and allows them to meet and interact with peers during a specific event prior to the main conference. It is a good experience for several European RSGs to coordinate and work together on a common event. The organizers of ECCB supported this initiative wholeheartedly and provided funding for several student travel fellowships that covered the costs to attend the entire conference. About 35 young scientists attended the first ESCS and were very pleased with the event. The interactive workshop on “Presenting Science Using the Theatre Approach” received an enthusiastic response. In working together to organize such an event, different RSGs can share their expertise, complement each other, and build on previous experiences.

### Benefits and Challenges

The most daunting challenges conference-organizing committees have to face are tasks that are so labor-intensive that they require teamwork, and individuals in the committee are expected to respond to requests efficiently. (i) Time management, (ii) financial support, and (iii) public relations are the three largest challenges for students planning to set up international meetings.

- As most organizers are students or postdocs with research and teaching obligations, they often have limited time available for these activities. Organizers should break down all responsibilities into manageable tasks with defined results in order to distribute the workload on many shoulders. It is easy to find volunteers for well-defined, specific tasks with calculable durations. Borrowing formalized project plans from other groups and asking past organizers to hand down their knowledge is the best recipe to follow for the new organizing committee.
- Students often lack the necessary professional standing to obtain financial support from companies or their institution. As such, it is difficult to publicize the event and obtain compensation for travel expenses. However, high-profile scientists can pay their own way, and a determined volunteer is usually able to find some financial support from industry sources. In some cases, faculty advisors or RSG members' supervisors can be convinced to help out with obtaining institutional resources.
- Communication is one of the major issues that surfaces in many interesting forms. Many non-native speakers find professional communication in English daunting; they require encouragement to participate and improve their skills. Effective communication is also necessary in getting the word out about an event. RSG members can be informed about an event via the RSG mailing list, and they will even help out by forwarding the announcement and spreading the word at their local institution if asked to do so. Furthermore, cross-border collaboration is accelerated through the use of social media and web-based project-management tools. These means alleviate the public relations challenges.

## Conclusion

In conclusion, this article highlights that there is strength in unity. Working together on common initiatives is often challenging but will also be extremely rewarding for both the attendees and the organizers. Bringing more diversity to events will make them richer. In the age of novel communication platforms, the number of interactions and collaborations are exploding. With the help of modern tools and effective best-practices, there is no reason why your group's next event shouldn't be international. Let's cross borders for science.

**About the Authors**

The authors have been involved in several aspects of the ISCB Student Council and its Regional Student Groups. **Sebastian J. Schultheiss** was president of RSG-Germany (2009–2012). **Joshua SungWoo Yang** was a cofounder and president of RSG-Korea (2007–2011) and general chair of AYRCOB (2011). **Wataru Iwasaki** was a cofounder and secretary of RSG-Japan (2009–2011). **Shu-Hsi Lin** was a cofounder and secretary of RSG-Taiwan (2011–2013). **Angela Jean** was a cofounder and president of RSG-Singapore (2007–2011) and general chair of AYRCOB (2010). **Magali Michaut** was cofounder and president (2008–2010) of RSG-France and served on its Board of Directors (2008–2013). She was also cofounder, secretary (2009), and president (2010–2011) of RSG-Europe and served as secretary for the ISCB Student Council (2009).
